# Mississippi Valley Dental Association

**Published:** 1873-10

**Authors:** Frank A. Hunter


### Proceedings of Societies.

### MISSISSIPPI VALLEY DENTAL ASSOCIATION.

The 29th annual meeting of the Mississippi Valley Dental Association was held in the lecture room of the Ohio Dental College, in Cincinnati, on Wednesday, March 5, 1873. The meeting was called to order by President Rehwinkel, and opened by prayer by Dr. James Taylor, after which the roll was called and the minutes of the 28th annual session read by the secretary, which were approved.

The Executive Committee made the following report which was acted upon and adopted by the Association :

ORDER OF BUSINESS.

1st. Calling of the Roll.

2d. Reading of the Minutes.

3d. Reports of Standing Committees—Executive Committee—Committee on Membership—Committee on Ethics.

4th. Reports of Special Committees.

5th. Election of Members and Payment of Dues.

ESSAYS AND DISCUSSIONS.

Subjects—1st. Dental Legislation : Is it practicable ? What benefits to the public and profession may be expected by enforcing such laws ?

2d. Artificial Dentistry: Should it be practiced as a specialty ? What may be done to raise the standard of operations in this department ?

3d. Filling Teeth : What may the patient reasonably expect shall be accomplished by the operation ?

4th. Methods and materials most suitable to obtain the best results in filling teeth.

5th. Dr. Arthur's method of treatment and prevention of decay of teeth.

6th. Conservative treatment of exposed or diseased dental pulps.

2th. Diseased condition of the soft tissues of the mouth—their pathology and treatment.

8th. Dental Fees. What rules should govern the Dentist in estimating the value of his professional services?

9th. The Dental Office—Ventilation—Light—The use of Magnifying Lenses.

10th. The use of mechanical power in operative dentistry.

Members are earnestly requested to prepare essays and papers to be read at this meeting. Those relating to the question under discussion will be read upon the introduction of the subject.

H. A. SMITH,<br>

I. KNAPP,<br>

W. P. HORTON. } *Executive Committee.*

Upon motion, it was decided to set apart one hour of the afternoon session of the 2d day for the exhibition of appliances.

On motion it was Resolved, that the secretary notify all members in arrears for dues, and that they shall be considered members in full standing upon the payment of the same.

A committee consisting of J. Taft and A. Berry was appointed to revise the roll.

A committee consisting of H. R. Smith, J. Taft and D. W. Clancey was appointed to consider the propriety of holding the meeting in College Hall, and giving a general invitation to the profession and public to attend.

The committee on membership reported the following names: Seneca B. Brown, Ft. Wayne, Ind.; B. Oscar Doyle, Louisville; J. W. Jay, Richmond, Ind.; James Leslie, Cincinnati; W. H. Eames, St. Louis; J. T. McMillan, Paris, Ky.; W. P. Hall, Piqua, Ohio; S. E. Goe, Cincinnati; H. W. Howe, Chillicothe, O.; all of whom were elected.

It was resolved that the time of meeting should be from 9 to 12, 2 to 5 1-2, and 7 1-2 to 10.

A dispatch from G. W. Keeley, announcing the death of Dr. Rosson, of Troy, O. was received and acted upon with becoming respect; and a committee consisting of Drs. McKellops, Taft and Hunt was appointed to draft suitable resolutions in reference to the same.

Prof. Eames having stated that C. S. Tomes, of London, was on his way west—he was instructed to dispatch to him extending the hospitality of this Association, and a committee of five was appointed to receive him.

An auditing committee was appointed consisting of Drs. Will. Taft, Hunter and Wheeler.

By consent of the Ex. committee the first subject for discussion was postponed until the morning of the 2d day. Adjourned.

WEDNESDAY, P. M.

Meeting called to order at 2:20. Minutes of morning session read and approved. Committee on College Hall reported through Dr. Taft. Report received and committee discharged.

The 2d subject for discussion—Artificial Dentistry. Should it be practiced as a specialty? What may be done to raise the standard of operations in this department?—was opened by the reading of a paper by Dr. Frank A. Hunter, and continued by Drs. H. R. Smith, J. Taylor, Taft, Hunter, Osmond, Harroun, Eames, H. A. Smith, Horton, McKellops, Berry, Jay, Knapp, Hall and Leman. The president's address relating somewhat to the subject under discussion was read at this place, and on motion a committee, consisting of Drs. Eames, Horton and Knapp, was appointed to take into consideration the universal appropriation of the title Doctor, and if in their judgment, make a report setting forth the sense of this meeting.

On motion, it was decided to dispense with the evening session. Adjourned.

THURSDAY, A. M.

The meeting was called to order by the president at 9 1-2, and after prayer by Dr. Leslie the secretary read the minutes of the last session, which were approved.

A dispatch from Dr. Morgan was read stating his inability to attend the meeting on account of a railroad accident; being painfully, but not dangerously injured.

On motion, the 3d subject, Filling teeth: what may the patient reasonably expect shall be accomplished by the operation? was taken up instead of the first, and was discussed by Drs. Taft, H. A. Smith, Osmond, Hunter, Knapp, Harroun, Rehwinkel, Wells, Palmer, Taylor and Horton—the subject by this time being about exhausted was declared closed, and the first subject, Dental legislation : Is it practicable ? What benefits to the public and the profession may be expected by enforcing such laws ? was opened by the reading of a paper by Dr. Osmond, which, not pertaining to the subject, was referred back to the author, after which the subject *proper* was discussed by Drs. H. A. Smith, Taft, Harroun, H. R. Smith, Rehwinkel and Horton.

Mr. Hamilton was requested to give his views on Dental Legislation, but wishing some little time to prepare himself and 12 o'clock having arrived, it was decided to hear him at 3 o'clock P. M. Adjourned.

THURSDAY, P. M.

One hour of this session being devoted to the exhibition of appliances, the meeting was not called to order until 3 o'clock. The minutes of the morning session were read and approved, and then Mr. Hamilton, of Washington, Ohio, took the floor and gave his objections to the Dental Law in Ohio, stating that it would be more generally supported if modified so as to include those who had been engaged in the practice of dentistry for a specified time. His remarks caused considerable discussion, during which a dispatch from Dr. S. S. White, of Philadelphia, in regard to the Gardner suit, was read, and received by three cheers for S. S. White.

By general consent a dispatch in answer was sent to Dr. White.

On motion, Dr. Palmer was requested to exhibit and explain his dental instruments, which occupied the balance of the session. Adjourned.

THURSDAY EVENING SESSION.

The meeting was called to order at 7 1-2 o'clock by the Pres. Minutes of the afternoon session read and approved, and then the fourth subject : Methods and materials most suitable to obtain the best results in filling teeth, was taken up and opened by the reading of a paper, by Dr. Stipp.

Prof. Eames, of St. Louis, was requested to describe his experiments in gutta percha, after which a letter was read by the Sec. from Prof. C. M. Wright, of Basel Switzerland.

Miscellaneous business having crept into the meeting, on motion of Dr. McKellops, it was resolved to hold our next meeting in the city of St. Louis, Mo.

Dr. Cameron, the Treasurer, made his report, which was referred to the auditing committee.

The following bills were presented and ordered to be paid :

Frank A. Hunter, advertising 29th annual meeting, Miss. Val. Ass., $5 50 ; J. Taft, telegraphing, $4 00 ; W. H. Eames, telegraphing, $4 10.

The committee on the universal appropriation of the title of Doctor made the following report, which was accepted, and committee discharged.

WHEREAS, The attention of this society has been called to the fact, that many persons in this and other states, have been in the habit of and now are using the prefix Doctor to their names, without having had the degree of Doctor conferred on them, neither by a dental or a medical college, and

WHEREAS, The use of said prefix without authority, is misrepresenting the actual standing of the persons so using it, and unjust to those who have earned the right lawfully to use it ; therefore

*Resolved*, That the members of this society most earnestly but respectfully remonstrate against the improper use of the prefix Doctor by dentists, and pledge themselves not to use any title not lawfully belonging them.

W. P. HORTON,

J. KNAPP,

W. H. EAMES.

} *Committee.*

The 4th subject having been exhausted, on motion, the 6th subject, Conservative treatment of exposed diseased dental pulps, was taken up and discussed by Drs. Hunt, Rawls, Eames and Hunter. On motion, it was decided to continue the discussion the next day. Adjourned.

FRIDAY A. M.

Meeting called to order by the Pres. at 9:15 and after prayer by Dr. Leslie, the Secretary read the minutes of last session which were approved. On motion, the Recording Secretary was instructed to enter upon the minute book the different resolutions and reports of committees.

The 6th subject was continued by Drs. H. A. Smith, McCullom, Knapp, Taylor, Rehwinkel, Hunter, Brown, Stipp, Berry, Clancey and Bradley.

The time having arrived, the Association proceeded to the election of officers for the ensuing year, which resulted as follows:

*President*,—W. H. Eames, St. Louis, Mo;

1*st*. *Vice President*,—S. B. Brown, Ft. Wayne, Ind;

2*nd*. " " H. R. Smith, Cincinnati;

*Recording Secretary*,—Frank A. Hunter, Cincinnati;

*C rresponding Secretary*,—A. F. Emminger, Columbus;

*Treasurer*,—J. G. Cameron, Cincinnati.

The Association sent a dispatch to Columbus remonstrating against repealing or changing the Ohio Dental Law.

The Committee on membership reported the names of F. A. Wernett and D. C. Hawxhurst, both of whom were elected.

The Auditing Committee reported on the Treasurer's report of 1870, 71 and 72, finding the same correct the Committee was discharged.

The Committee on Appliances made the following report, which was accepted and the Committee discharged:

The Committee on Appliances have had presented to them for consideration crystalline gold, manufactured by James Leslie; it is highly recommended by those who have used the same, and they think it worthy of a trial.

We have also had presented to us for consideration, a pocket drill, by Dr. Baxter, which seems to be very ingenious and convenient for those that cannot afford a more expensive one, and yet it is so made, that it may be applied to other machines running with a cord, but it is wanting in steadiness. Dr. Baxter also has presented some points for polishing, made from Arkansas stone, which seem to meet a "want long felt."

We have not time to give the details of any of the above.

MERRIT WELLS,<br>C. PALMER,<br>R. A. MOLLYNEAUX. } *Committee.*

On motion, the officers elect were to be installed at 2 P. M. Adjourned.

FRIDAY P. M.

Meeting called to order by President at 2:30, after which the Secretary read the minutes which were approved. Prof. Eames, the President elect, was conducted to the chair, and after a few remarks by Dr. Rehwinkel (the retiring President) cordially thanked the Association for the honor conferred upon him, and complimented the Association in such terms, that your Secretary blushes to record.

The 6th subject being very popular, was *again* taken up and discussed by Dr. Taft, H. A. Smith, Hunter and Palmer.

The President announced the following Committees for the ensuing year:

*Executive Committee.*—W. N. Morrison, Mo.; F. H. Rehwinkel, Ohio; P. G. C. Hunt, Ind.

*Committee on Membership.*—W. H. Goddard, Ky; G. W. Keeley, Ohio; D. C. Hawxhurst, Mich.

*Committee on Ethics.*—W. H. Morgan, Tenn.; J. Taft, Ohio; H. J. McKellops, Mo.

*Committee on Appliances.*—H. McCullom, Ky.; D. W. Clancey, Ohio; J. W. Jay, Ind.

The following were elected Delegates to the American Dental Association:

I. Knapp, D. C. Hawxhurst, J. A. Stipp, D. W. Clancey, B. D. Wheeler, James Leslie, J. W. Jay, S. E. Goe, D. W. Roudebush, W. F. Harbaugh, H. W. Howe, Frank A. Hunter, C. Welch, Merrit Wells, H. McCullom and C. Bradley.

After ordering the usual bills to be paid, the Association adjourned to meet in St. Louis in March, 1874.

FRANK A. HUNTER, *Recording Secretary.*

